# Prognostic models based on imaging findings in glioblastoma: Human versus Machine

**DOI:** 10.1038/s41598-019-42326-3

**Published:** 2019-04-12

**Authors:** David Molina-García, Luis Vera-Ramírez, Julián Pérez-Beteta, Estanislao Arana, Víctor M. Pérez-García

**Affiliations:** 1 Mathematics Department, Mathematical Oncology Laboratory (MôLAB), Universidad de Castilla-La Mancha, Ciudad Real, Spain; 2 Helmholtz-Zentrum Berlin, Berlin, Germany; 3 Radiology Department, Fundación Instituto Valenciano de Oncología, Valencia, Spain

## Abstract

Many studies have built machine-learning (ML)-based prognostic models for glioblastoma (GBM) based on radiological features. We wished to compare the predictive performance of these methods to human knowledge-based approaches. 404 GBM patients were included (311 discovery and 93 validation). 16 morphological and 28 textural descriptors were obtained from pretreatment volumetric postcontrast T1-weighted magnetic resonance images. Different prognostic ML methods were developed. An optimized linear prognostic model (OLPM) was also built using the four significant non-correlated parameters with individual prognosis value. OLPM achieved high prognostic value (validation c-index = 0.817) and outperformed ML models based on either the same parameter set or on the full set of 44 attributes considered. Neural networks with cross-validation-optimized attribute selection achieved comparable results (validation c-index = 0.825). ML models using only the four outstanding parameters obtained better results than their counterparts based on all the attributes, which presented overfitting. In conclusion, OLPM and ML methods studied here provided the most accurate survival predictors for glioblastoma to date, due to a combination of the strength of the methodology, the quality and volume of the data used and the careful attribute selection. The ML methods studied suffered overfitting and lost prognostic value when the number of parameters was increased.

## Introduction

Glioblastoma (GBM) is the most common and lethal malignant primary brain tumor with the worst prognosis. A substantial amount of research has been devoted to understanding different aspects of the disease, specifically the development of different types of biomarkers. Clinical, molecular and imaging parameters have been used to build mathematical models able to classify GBM patients in terms of survival, identify GBM subtypes, predict response to treatment, etc^1–3^.

Machine learning (ML) techniques have been increasingly used by the radiological research community^4,5^ to construct such models^6,7^. These methods, when used on sufficiently large data sets, are able to extract hidden information and patterns from data, automatically learning and being able to make predictions about future system behavior.

ML remains a young field with many underexplored research opportunities^8,9^. The application of ML in radiology, typically being based on large sets of features extracted from medical images, and known as radiomics^1,4^, has a great potential to increase clinical efficacy. However, together with several interesting applications and discoveries, there have been many studies with serious experimental design flaws^8,9^. Most pitfalls of ML methods in biomedical research result in common problems such as overfitting^8,9^.

In this study we developed efficient, optimized predictive models using clinical data and high-quality MRI-based morphological information of GBM patients. First, we developed a human-built optimized linear predictive model (OLPM) on the basis of the researchers’ understanding of the predictive value of the variables showing individual prognosis value. We also constructed ML models using some of the best methods available: artificial neural networks (ANN), support vector machines (SVM), and regression trees (RT). Our intention was to compare the OLPM with the ML approaches and the best ML-based models recently proposed in the literature to construct accurate prognostic estimators and to show how a non-rigorous use of ML methods in neuro-oncology can lead to misleading results.

## Results

### Kaplan-Meier and Spearman correlation analyses

Parameters achieving marginal statistical significance in the Kaplan-Meier analysis for the discovery cohort were age, CE volume, CE rim width, maximum tumor diameter and surface regularity. Thresholds which best split the sample into significant subsets in terms of c-index were: 65 years for age (p-value < 0.001, HR = 2.086, c-index = 0.706), 18 cm^3^ for CE volume (p-value = 0.062, HR = 1.250, c-index = 0.556), 0.416 cm for CE rim width (p-value = 0.013, HR = 1.378, c-index = 0.590), 5.0405 cm for maximum tumor diameter (p-value = 0.063, HR = 1.246, c-index = 0.572) and 0.509 for surface regularity (p-value = 0.002, HR = 1.476, c-index = 0.578). No other volume or surface-based parameter obtained marginally significant results in the Kaplan-Meier analysis.

Age, CE rim width and surface regularity showed no correlation with the marginally significant parameters obtained. However, CE volume and maximum tumor diameter were strongly correlated (Spearman correlation coefficient = 0.916, p-value < 0.001), so the latter was discarded when building the OLP model.

### Optimal linear prognosis model

A multivariate Cox regression model was constructed using age, CE volume, CE rim width and surface regularity. This model gave c-indexes of 0.735 in the discovery cohort and 0.744 in the validation cohort.

Then, 30^4^ linear predictive models were constructed using the procedure explained in the Methods section. The equation of the PS of the best linear model including age, CE rim width, surface regularity and CE volume was

$$\begin{array}{c}OLPM={0}.{030}\cdot age-{0}.{340}\cdot CE\,rim\,width-{1}.{100}\cdot surface\,regularity\\ \,\,\,\,\,\,+{0}.{012}\cdot CE\,volume.\end{array}$$

**Figure 1:**
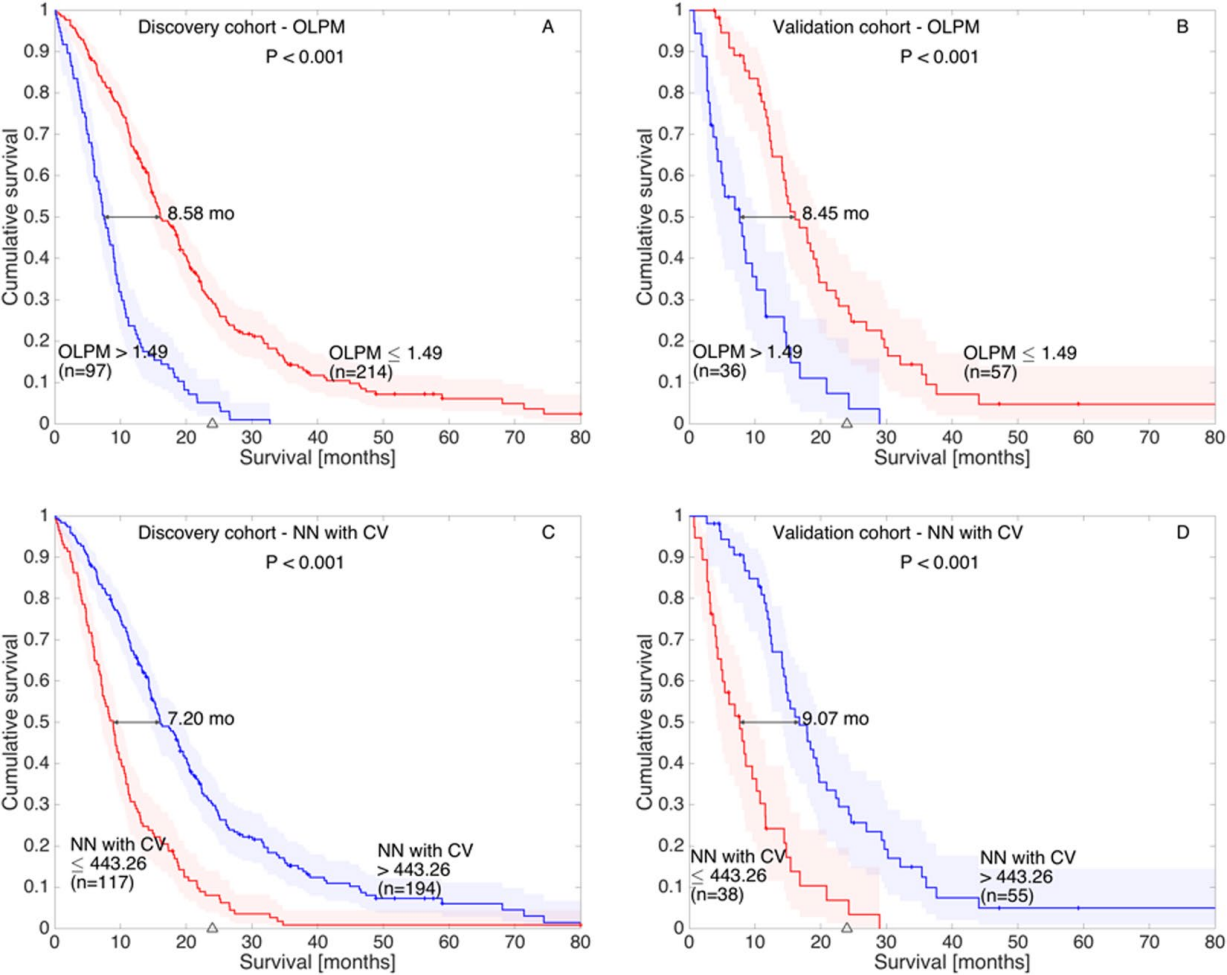
Kaplan-Meier curves obtained for the OPML and the best ML method (NN with CV) in the discovery (**A**,**C** respectively) and validation (**B**,**D** respectively) cohorts.

The c-indexes obtained for this model were 0.771 on the discovery cohort, and 0.817 on the validation cohort. Table 1 summarizes the best results obtained by each algorithm in both cohorts and Fig. 1 shows the Kaplan-Meier curves obtained using the best threshold (1.49) in the discovery (Fig. 1A) and validation (Fig. 1B) cohorts.

**Table 1:** Summary of the performance and number of attributes used for the different models studied in this paper.

Model	Number of parameters	c-indexes	
		Discovery	Validation
Cox	4	0.735	0.744
Best linear (OLPM)	4	0.771	0.817
NN with CV	4	0.791	0.825
NN	4	0.740	0.751
RFF_SVM	4	0.747	0.783
libSVM	4	0.739	0.756
RT	4	0.696	0.681
NN	44	0.794	0.746
RFF_SVM	44	0.801	0.766
libSVM	44	0.752	0.700
RT	44	0.741	0.630

Cox, best optimized linear prognosis model and machine learning-based approaches are included. Results are listed for both the discovery and validation cohorts. C-indexes in the validation group over 0.8 are boldfaced.

### ML models

The NN constructed using age, CE volume, CE rim width and surface regularity as parameters obtained c-indexes of 0.740 and 0.751 in the discovery and validation cohorts respectively. However, equipped with all the 44 parameters, NN obtained c-indexes of 0.794 and 0.746 respectively.

The optimal attribute combination extracted from the CV process included age, surface regularity, total surface and CE volume. It presented an average c-index along the 20-fold CV (with 50 test patients) of 0.791. This model configuration, trained with the entire discovery cohort and applied with the same threshold on the validation cohort, obtained a c-index of 0.825. Figure 1 shows the Kaplan-Meier curves obtained by this model (NN with CV) in the discovery (Fig. 1C) and validation (Fig. 1D) cohorts.

The libSVM method, when restricted to the four morphological parameters, obtained c-indexes of 0.739 and 0.756 in the discovery and validation cohorts respectively. The non-restricted instance of this algorithm achieved c-indexes of 0.752 and 0.700 respectively.

The RFF_SVM algorithm, when based on the four marginally significant parameters in the Kaplan-Meier analysis, obtained a c-index of 0.747 in the discovery cohort and 0.783 in the validation cohort. The use of all parameters led to c-indexes of 0.801 and 0.766 respectively.

Finally, the RT model using age, CE volume, CE rim width and surface regularity as parameters obtained c-indexes of 0.696 and 0.681 in the discovery and validation cohorts respectively. However, equipped with all the 44 parameters, it obtained c-indexes of 0.741 and 0.630 respectively. Table 1 summarizes these results and compares the performance of the ML and OLP models.

**Figure 2:**
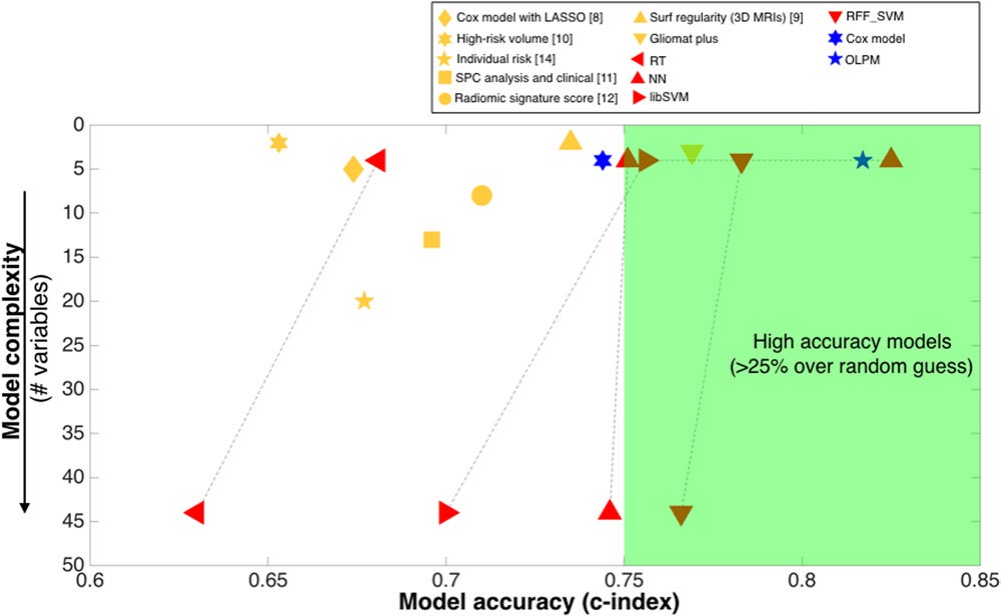
Comparison of the predictive value (c-index) and number of variables for the models developed in this paper versus representative models from the literature. Previous approaches are shown in yellow, with different symbols corresponding to different studies. ML methods described in this paper are shown in red and linear models in blue. Results are given for the best models in each reference and for the validation groups when available.

Figure 2 summarizes the results of our prognostic models (linear and machine learning-based models) in comparison with other recent approaches.

## Discussion

Our study has implications for clinical practice. The development of prognostic models in GBM has attracted substantial attention in recent years^1,3,10^. Prediction models are typically constructed by combining the information available from patient clinical data, laboratory and imaging data. However, their merger into effective predictive models is not trivial and most of the approaches followed are based on ML methods. The experience from this preliminary derivation suggests that a parsimonious list of MRI features -CE rim width, CE volume and surface regularity- and age could be sufficient for routine clinical use. Besides simplicity, it discards a large number of imaging findings, only attainable with laborious post-imaging, post-processing procedures.

The use of ML methods to develop statistical models for prognosis, prediction and classification in biomedical research is growing steadily^1,4,8,9,11^. The broad popularity of these methods is due to a combination of factors: first, their relative objectivity and ease of integration in the research once the data has been extracted. Secondly, in biomedical research, there are numerous variables and subjective decisions that must be taken into account, causing differences between professionals which may be resolved by ML methods^5^. Finally, the availability of commercial software packages has made the use of this kind of technique easier, even for researchers without ML knowledge^9^. However, these methods are often used as “black boxes”, especially ANN and SVMs, making them prone to finding spurious associations between meaningless parameters. This is why some research training in these techniques is necessary in order to use them correctly. In addition, all findings obtained from ML methods must be validated on independent datasets^5,8,9,11,12^.

The key ingredient of all ML methods is data, but data is useless until information and knowledge are extracted from it^12^. Thus, researchers must only use a widely-refined dataset, and ML algorithms must be fed with parameters containing medically relevant information^9^. Radiomic-based studies are too often characterized by the use of thousands of parameters obtained from clinical practice, imaging, genomics, etc. from a reduced patient dataset^1,2,6,8,9^. This data is put together into the ML engine, which is supposed to both discard the meaningless parameters and construct a classifier with the remaining ones. However, ML methods have many limitations when performing such tasks, especially with small-sized data sets, and these tasks must be addressed in order to develop meaningful models^9,11^. Thus, it is of enormous importance in to limit the number of parameters to those bearing information about the disease, since inadequate data sources and modeling processes lead to methodologically inadequate studies^9,11^. For this reason, simple and rigid models, such as statistical linear regression models based on human knowledge, must not be forgotten, due to their proven reliable findings and rigid designs, less susceptible to mistakes^12^.

In this study, we used a discovery database of 311 local GBM patients with available pretreatment volumetric postcontrast T1-weighted MRIs, constituting one of the largest series of GBM patients with pretreatment volumetric data in the literature^13–15^. Also, we used a database of 93 patients from public databases (TCIA) as a validation cohort, including only patients with available pretreatment volumetric MRIs. The segmentation of all these patients was performed semi-automatically, a time-consuming procedure, in order to obtain data of the highest possible quality. Clinical data was carefully obtained and revised^16,17^. We built prognostic models for GBM patients using both a simple statistical methodology based on a small set of meaningful variables, and also ML methods. We developed a linear model (OPML) with similar discriminatory capability to ML methods and without significant differences between them. We performed narrow statistical validation of these models and revealed which predictors could be omitted without loss of predictive power.

Morphological MRI-based features have been identified in the literature as key biomarkers for overall survival in GBM^16–18^, in addition to clinical parameters such as age or extent of resection, broadly known to have high predictive value in GBM^3,19–21^. Many recent papers have employed MRI-based texture data to obtain hundreds of parameters to feed ML models^6,9^. Although those parameters are strongly dependent on MRI protocols and image resolutions^22,23^, several articles have accomplished excellent performance in the identification and/or classification of tumor characteristics or patient survival^1,2,9,12,14,16,17,24^.

To construct the linear predictive models, we only used morphological features coming from biologically-inspired mathematical models^25^ whose prognostic value had been demonstrated on previous versions of the same database, i.e. having either fewer patients or less up-to-date survival data^16,17,26^. In addition, we considered other morphological surface and volume-based features whose prognostic value has been controversial in the literature, such as total tumor volume, necrotic tumor volume, tumor surface, and tumor diameter^14,27^.

We also included in the experimental phase ML methods to construct predictive models. Specifically, we tested an ANN, two SVMs and a RT. In order to fit out the ML methods with more data, we added texture-based parameters computed using gradient variations and co-occurrence and run-length matrices. Dozens of imaging parameters have been proposed in the literature for classification purposes of GBM survival^1,2^. Although further tuning of the parameters would be interesting, for our purposes we restricted our set of parameters including only the most popular textural parameters in the literature^1,2^.

Regarding accomplished results, it is notable that the OLPM, built on the basis of age and the morphological variables (CE rim width, surface regularity and CE volume), achieved c-indexes of 0.771 for the discovery cohort and 0.817 for the validation cohort. None of the ML methods, when fed with this specific set of parameters, was able to surpass these results. Only the optimal combination extracted from the CV process for the ANN (0.791 as mean c-index on the discovery cohort over the CV and 0.825 on the validation cohort) obtained comparable results. This is a good example of the hugely important role that feature selection plays in model accuracy. In fact, the attributes extracted with the Kaplan-Meier analysis were probably optimal for the linear regression but not for the non-linear regression carried out by the ANN, which needed a specific CV process in order to be able to obtain comparable results.

On the other hand, in order to show how saturated databases may corrupt the results obtained with ML methods, we also considered the use of ML methods with the full set of morphological plus textural variables. Then, as might be expected, some of the ML methods, specifically the NN (0.794) and the RFF_SVM (0.801), obtained better results than the OLPM (0.771) on the discovery cohort. Having so many degrees of freedom allowed the ML methods to learn the discovery dataset more accurately. However, those results were not reproduced on the validation cohort. Instead, the OLPM (c-index = 0.817) outperformed all the ML approaches (c-indexes of 0.746, 0.766, 0.700 and 0.630).

Similarly, it is worth mentioning that the ML methods obtained a higher c-index in the discovery cohort using the full parameter set as opposed to only using the four outstanding prognostic parameters. However, results for the validation cohort were the opposite: every ML method obtained a higher c-index using the model constructed with only 4 parameters. This is a clear indication that results obtained by the models using the 44 parameters led to some level of overfitting.

For comparison, in the last few years many studies have constructed prognostic models for GBM patients, most using ML techniques^13,14,17,26,28,29^. The best prognosis indicators developed using only clinical variables achieved a c-index of only 0.582 in the validation cohort^15^. When adding imaging features results were greatly improved in many studies. Cui *et al*.^28^ obtained 0.653 with two parameters and 0.674 using five imaging features^13^ for their validation cohorts. Ingrisch *et al*.^14^ obtained a c-index score of 0.677 in their CV analysis using a model based on 20 parameters. Lao *et al*.^29^ obtained a c-index of 0.710 in their validation dataset using 8 features. We have previously developed linear prognostic models using age and surface regularity^17^ (c-index of 0.735) and later adding the CE rim width (c-index of 0.769)^26^. It is interesting to note that the OLPM and the ML-based models developed in this study surpassed all previous reported ML-based results, and also the well-designed methodology of the algorithms and the reliability of the data used.

Data science, and so ML, is becoming an important subject and it has a promising future in biomedical research. However, ML methods should be used carefully and within their range of applicability to avoid false conclusions. It is especially relevant to avoid the use of hundreds of meaningless features on data coming from limited numbers of patients, in most cases less than a hundred. Thus, there is room for simpler human-built models based on parameters underlying proven information about the disease which in this study showed equivalent accuracy and lower computational costs than approaches coming from ML techniques.

The study has several strengths worth mentioning. Firstly, it encompasses one of the largest series of GBM patients with pretreatment volumetric postcontrast T1-weighted MRIs in the literature^13–15^. Secondly, it provides reliable clinical practice data^27^, where limitations of clinical trials are commonly encountered^15^. Third, all segmentations were performed manually, reviewed by experienced radiologists and the morphological measures were always computed in 3D. Finally, all results were validated using a large and public dataset of patients from TCIA^30^.

Regarding the limitations, the first is the lack of genetic and molecular information and recurrences on the cohorts due to the retrospective nature of the study, which would probably have led to even better survival predictions. Secondly, only pretreatment volumetric postcontrast T1-weighted MRIs were used to characterize morphological tumor properties. Although this sequence has shown high discriminating power^13,31^, other image sequences may be included in future research. Also, lack of uniform imaging acquisition on the remaining sequences available in the study could hamper meaningful image segmentation and comparison of results. Thirdly, given the multicenter nature of the study, and although great effort was made to homogenize data, there were differences in the imaging protocols and clinical follow-up.

In conclusion, OLPM and ML methods studied here provided the most accurate prognostic predictors for glioblastoma to date. This was the result of a combination of the strength of the methodology, the quality and volume of data used and the careful attribute selection. The ML methods studied suffered from substantial overfitting and lost prognostic value when the number of parameters was increased.

ML has the potential to change clinical neuro-radiology. Our study shows that: (i) a correct implementation of ML methods is essential to avoid obtaining conclusions of limited validity and (ii) other human approaches based on careful understanding of the mechanistic behavior behind the tumor growth processes may provide competitive prognoses and predictive measures of the disease.

## Materials and Methods

### Patients

The study was approved by the Institutional Review Board of Fundación Instituto Valenciano de Oncología, and then implemented in the ten participating local medical institutions. All experiments were performed in accordance with relevant guidelines and regulations. Informed consent for study participation was obtained for each patient included.

A total of 404 GBM patients were included in the study. Of these, 311 patients (62 ± 12 years old, 44% females and 56% males) were retrospectively selected from an initial set of 1155 patients from ten local medical institutions in the period 2006–2017. Inclusion criteria were: (i) pathologically proven GBM according to the 2007 World Health Organization (WHO) Classification of Tumors of the Central Nervous System, (ii) unifocal lesions, (iii) availability of relevant clinical variables: age, type of resection performed, and survival information at last follow-up, (iv) availability of a pretreatment volumetric CE T1-weighted MR imaging sequence (slice thickness ≤2.00 mm, spacing between slices ≤2.00 mm, no gap, pixel spacing ≤1.20 mm), (v) no substantial imaging artifacts, and (vi) presence of contrast-enhancing areas. Survival of censored patients included in the study was updated up to February 2018.

Gross total resection was defined as the absence of visible nodular tumoral enhancement after surgery as determined from the CE T1-weighted MRI within 72 hours after surgery. Overall survival (OS) was computed from the date of the preoperative MRI until death or last follow-up examination. The latter were considered as censored events.

A validation cohort was obtained from the public repository: The Cancer Image Archive (TCIA)^30^. We reviewed 431 patients from the TCIA databases: 262 from the The Cancer Genome Atlas for glioblastoma (TGCA-GBM)^32^, 130 from REpository for Molecular BRAin Neoplasia DaTa (REMBRANDT)^33^ and 38 from Ivy Glioblastoma Atlas Project (IvyGAP)^31^. Of these, 93 patients (63 ± 14 years old, 47% females and 53% males) met the inclusion criteria described above. Table 2 summarizes the main patient characteristics for the different patient cohorts and Supplementary Section S1 shows the list of TCIA patients included in the study.

**Table 2:** Summary of patient characteristics, MR imaging and volumetric parameters for the groups of patients considered in the study.

		Discovery cohort	Validation cohort
Patient characteristics	Number of patients (censored)	311 (27)	93 (12)
	Age (years) median (range)	63 (19–86)	62 (14–86)
	Sex {male (M), Female (F)}	44% F; 56% M	47% F; 53% M
	Survival (months) median (range)	12.76 (0.13–82.97)	11.77 (0.72–59.20)
	Type of resection (total, subtotal or biopsy)	149 Total (47.91%)	17 Total (18.28%)
		113 Subtotal (36.33%)	—
		49 Biopsy (15.76%)	8 Biopsy (8.60%)
		—	68 Unknown (73.12%)
	Type of treatment (Chemotherapy (CT) and Radiotherapy (RT))	241 CT + RT (77.49%)	74 CT + RT (79.57%)
		27 RT alone (8.68%)	4 RT alone (4.30%)
		5 CT alone (1.61%)	4 CT alone (4.30%)
		38 no treatment (12.22%)	11 no treatment (11.83%)
MRI characteristics	Pixel spacing (mm) mean (range)	0.81 (0.46–1.09)	0.90 (0.45–1.06)
	Slice thickness (mm) mean (range)	1.48 (1.00–2.00)	1.41 (0.90–2.00)
	Spacing between slices (mm) mean (range)	1.10 (0.50–2.00)	1.36 (0.70–2.00)
	Number of slices mean (range)	174 (80–360)	150 (72–305)
Volumetric parameters	Tumor volume (cm^3^) mean (range)	33.14 (0.48–132.54)	41.82 (2.47–116.12)
	CE volume (cm^3^) mean (range)	19.64 (0.44–90.06)	24.90 (2.46–90.95)
	Necrotic volume (cm^3^) mean (range)	13.50 (0.03–89.31)	16.92 (0.00–69.20)
	CE rim width (cm) mean (range)	0.57 (0.22–1.65)	0.63 (0.24–1.25)
	Maximum diameter (cm) mean (range)	5.11 (1.30–11.09)	5.71 (2.55–9.80)
	Total surface (cm^2^) mean (range)	67.27 (3.00–226.32)	83.13 (13.27–196.04)
	Surface regularity mean (range)	0.62 (0.24–0.99)	0.57 (0.30–0.83)

### Image acquisition

Postcontrast T1-weighted sequence was a gradient echo sequence using three-dimensional (3D) spoiled gradient-recalled echo or 3D fast-field echo, without magnetization transfer after intravenous administration of a single-dose of gadolinium (0.10 mmol/kg) with a (6–8)-min delay. All MR images were acquired in the axial plane.

The cohort from local institutions was imaged with scanners having magnetic field strengths of 1.5-T (n = 278) and 3-T (n = 33). The range of repetition times was 6–25 ms and 3–10 ms for echo times. General Electric (n = 136), Philips (n = 108) and Siemens (n = 67) scanners were used to obtain the images.

For the validation cohort from TCIA, magnetic field strengths of 1.5-T (n = 56) or 3-T (n = 37) were used, repetition times 6–34 ms and echo times 2–14 ms. General Electric (n = 46), Philips (n = 12) and Siemens (n = 35) scanners were used.

Other image and patient characteristics are summarized in Table 2.

### Image analysis

The DICOM files were analyzed using the scientific software package Matlab (R2017b, The MathWorks, Inc., Natick, MA, USA). Tumors were automatically delineated by the same image expert without normalization on the raw gray-level values, using a gray-level threshold chosen to identify the CE tumor volume. Then, the expert manually corrected each segmentation slice by slice. Necrotic tissue was defined as hypo-intense tumor regions inside CE tissue. An in-house software was developed allowing the segmentations to be corrected on a tablet using a digital pencil.

### Geometrical and textural measures

A set of 16 3D geometrical measures was extracted from the segmented tumors: these included total volume, CE volume, necrotic volume, CE rim width, maximum 3D diameter, total surface and surface regularity among others^16,17^. Also, a set of 28 3D textural variables containing gradient-based^34^, co-occurrence matrix (CM)^35,36^ and run-length matrix (RLM)^37,38^ features were computed from the segmented images. These measures provide a (local or regional) characterization of the gray-level variations between voxels within the tumor.

Supplementary Section S2 contains the full list of features and their mathematical expressions.

### Statistical analysis

Kaplan-Meier analysis was used to identify parameters associated with prognosis, using the Log-Rank and Breslow tests to assess the significance of the results. This method compares two populations separated in terms of one parameter and study their statistical differences in survival. For each parameter, we searched for every threshold value, splitting the sample into two different subgroups. Then, we chose as best the non-isolated significant value giving the lowest p-value^16,17,26^. A 2-tailed significance level of p-value lower than 0.05 was applied. After the threshold was set, the hazard ratio (HR) and its adjusted 95% confidence interval (CI) for each pair of subgroups was computed using a Cox proportional hazards regression analysis.

Spearman correlation coefficients were used to assess the dependences between pairs of variables. We considered significant correlation coefficients over 0.7 or below −0.7 to correspond to strong correlations between variables. Harrell’s concordance index score was also computed to evaluate the accuracy of the prognostic models^26,29,39^. This method compares the survival of two populations of patients (best prognosis versus worst prognosis) by studying all possible population between individuals belonging to different groups. Then, percentage of right guess is reported as result. Concordance indices were computed using the non-censored sample and ranged from 0 to 1, with 1 indicating a perfect model (a random guess would give a concordance index of 0.5). SPSS software (v. 22.0.00) was used for the statistical analysis.

### Construction of the OLPM

For the construction of our linear predictive models we considered only the morphological parameters, as textural parameters have been questioned recently due to their lack of robustness^9,22–24^.

Morphological parameters being marginally significant (p-value < 0.1) individually in the Kaplan-Meier analysis and non-correlated with other parameters were chosen to construct a linear prognostic model using multivariate Cox regression, the coefficients of the regression being labeled B_1,…,_ B_p_. These parameters were used as seeds for an algorithm searching for the OLPM. To do so, we constructed 30^p^ different models by analyzing the range (B_i_ − 25% B_i_, B_i_ + 25% B_i_) for each coefficient, with the interval discretized in 30 subintervals. For each model we searched for the population splitting for the discovery cohort providing the best c-index value. Finally, the best of the 30^p^ different models in terms of c-index was chosen as the OLPM.

### ML methods

The ML models used in this study were the following:

#### Artificial neural networks (ANN)

We work with a very simple fully-connected network with one hidden layer, whose number of neurons is given by the linear formula floor((n_features + 1)/2)^40,41^. The activation function of the hidden layer is the sigmoid, while the output layer gets a linear activation function. The network was trained with a simple SGD (200 iterations and learning rate 0.01) for backpropagation with momentum (0.5).

#### Support Vector Machines (SVM)

Two variations of this model were used:

(i) libSVM: implementation of the original SVM with Gaussian RBF Kernel from the library libSVM^42^. The model was trained with epsilon = 0.01 (with respect to normalized data), gamma = 1.0 and C = 0.001 (trade-off parameter).

(ii) RFF_SVM: Implementation based on^43^. Linearization of the original SVM model with Gaussian Kernel (gamma = 1.0) by a random sampling of its Fourier transform. The underlying feature space was set to 200 components, which allows flexibility in the number of input attributes. Optimized with SGD, epsilon = 0.01 (with respect to normalized data) and trade-off parameter 0.01. The chosen loss function is the squared epsilon loss.

#### Regression Trees (RT)

Classical implementation of a binary regression tree with variance-based splitting measure and pruning (50–50%)^10^. The tree was set with maximal depth 5 and a minimal decrease in measure of 0.1. In order to study the dependence and reproducibility of the results obtained by the ML methods, we firstly trained and evaluated these models with the data set filtered to the morphological parameters being marginally statistically significant in the Kaplan-Meier analysis.

After this, in order to show how overfitting can corrupt the learning process and the results of the ML models, we evaluated the methods including the full set of 44 parameters as attributes.

Finally, in order to build the best possible ML methods with a model-specific election of input attributes, we also analyzed attribute sets providing the best results for NN. To this end, we carried out a cross-validation (CV) process for attribute combinations extracted from a pre-filtered set of marginally-significant attributes. The pre-filter consisted in a feature selection based on random forests with cross-validation. The used random forest algorithm was the Scikit-learn implementation of the Extremely Randomized Trees (ExtraTreesRegresor) with the following non-standard parameters: number of estimator = 10, maximal tree depth = 5, minimal samples per leaf = 20, minimal samples for node split = 40, and minimal decrease of impurity = 0.1. Within this large CV (10000 iterations) the discovery cohort was randomly and repeatedly divided into train and test subsets, and a random forest successively trained and evaluated with respect to them. From each experiment we obtained weights for each attribute based on its Gini importance^44^. The final score for each feature is defined as the sum of the Gini importances of those random forests along the cross-validation whose validation root-mean-square deviation (RMSE) is at least 5% better than the baselines. The ranking of the final scores obtained by each attribute is outlined in Table S4. The attribute combinations presenting the best (mean) root-mean-square deviation (RMSE) among those with the best (mean) c-index were chosen for the final evaluation with respect to the validation cohort. The threshold taken for the validation cohort was the best obtained during the CV process.

Also, we performed a principal component analysis (PCA)^45^ in order to validate attribute selection. Basically, PCA is a statistical procedure which recursively reduces the global variance of the dataset by linear transformations that produce the so-called components (orthogonal and uncorrelated) and hence simplifies the complexity in high-dimensional data while retaining trends and patterns^45^. Table S5 shows the obtained PCA score matrix, in which the weight of patient survival starts being meaningful in component 6, in which age, surface regularity and contrast-enhancing rim width (the parameters selected in our model) also get large weights (in absolute value).

### Evaluation of the models

Both the OLP and ML models were constructed blindly using the discovery cohort. To evaluate the performance of the models built, we calculated the c-index for the independent validation cohort using the same model and threshold. This methodology ensured that the results obtained were robust, reproducible and independent of the data used to produce them^9.24^.

## Supplementary information

Supplementary material

## Data Availability

All data included in the article is available upon request to the corresponding author.
