# Supplementary material for: Prognostic models based on imaging findings in glioblastoma: Human versus Machine
Source: Sci Rep. 2019 Apr 12;9:5982. doi: 10.1038/s41598-019-42326-3 (PMC6461644; doi:10.1038/s41598-019-42326-3)
Supplement: Supplementary file 1 — Supplementary material [file 41598_2019_42326_MOESM1_ESM.pdf]

Prognostic models based on imaging findings in glioblastoma: Human versus Machine

David Molina-García* PhD, Luis Vera MSc, Julián Pérez-Beteta MSc, Estanislao Arana[$] MD PhD, Víctor M. Pérez-García[$] PhD.

$ Joint senior authors

Mathematics (DMG, JPB, VMPG) department, Mathematical Oncology Laboratory (MôLAB), Universidad de Castilla-La Mancha, Ciudad Real, Spain. Research Department (LV), prudsys AG, Berlin, Germany. Radiology (BA) department, Radiology (EA) department, Fundación Instituto Valenciano de Oncología, Valencia, Spain.

Supplementary material

Section S1

We list here the TCIA patients included in the validation group. From the REMBRANDT database: 900_00_5414 and 900_00_5458. From the TCGA database: TCGA-02-0034, TCGA-02-0048, TCGA-02-0059, TCGA-02-0060, TCGA-02-0064, TCGA-02-0070, TCGA-02-0075, TCGA-02-0106, TCGA-08-0244, TCGA-08-0348, TCGA-08-0350, TCGA-08-0353, TCGA-08-0354, TCGA-08-0355, TCGA-08-0357, TCGA-08-0358, TCGA-08-0360, TCGA-08-0385, TCGA-08-0389, TCGA-08-0392, TCGA-08-0509, TCGA-08-0510, TCGA-08-0516, TCGA-08-0518, TCGA-08-0521, TCGA-08-0522, TCGA-08-0529, TCGA-12-0616, TCGA-12-0620, TCGA-12-1094, TCGA-12-1098, TCGA-12-1600, TCGA-12-1602, TCGA-12-3646, TCGA-12-3648, TCGA-12-3650, TCGA-14-0817, TCGA-14-1043, TCGA-14-1402, TCGA-14-1454, TCGA-14-1459, TCGA-14-3477, TCGA-14-1825, TCGA-19-0955, TCGA-19-0964, TCGA-19-1385, TCGA-19-1386, TCGA-19-1387, TCGA-19-1389, TCGA-19-1392, TCGA-19-2623, TCGA-19-2625, TCGA-19-2629, TCGA-19-4068, TCGA-19-5951, TCGA-19-5952, TCGA-19-5955, TCGA-19-5958, TCGA-19-5959, TCGA-27-1833, TCGA-27-2523, TCGA-27-2526, TCGA-27-2527, TCGA-76-4928, TCGA-76-4935, TCGA-76-6191, TCGA-76-6193, TCGA-76-6280, TCGA-76-6285, TCGA-76-6656, TCGA-76-6657, TCGA-76-6663. From the IvyGAP database: W10, W11, W12, W13, W16, W18, W2, W20, W21, W29, W33, W34, W36, W38, W39, W40, W48, W5, W7,

Section S2

This section includes the description of the geometrical (16) and textural (28) measures considered in the study.

Geometrical measures considered

Volumes: The contrast enhancing volume (V_{CE}), necrotic (or inner) volume (V_I) and total volume ($V = V_{CE} + V_I$) were computed. These three measures were considered.

Contrast enhancing spherical rim width (δ_s)**:** It was defined as

$$\delta_s = 0.62[\sqrt[3]{(V_{CE} + V_I)} - \sqrt[3]{V_I}]$$

and measures the averaged width of the contrast enhancing areas by assuming sphericity of the necrotic volume and assuming that CE areas are placed peripherally with an annular shape. δ_s, its four quartiles, its median, its deviance, its maximum, its minimum and its mode values were considered.

Surface: The surface of the segmented tumor was computed using a triangularization based on the surface points and the *marching cube* method [22]. The surface of each connected triangle was computed independently and added for the computation of the total surface.

Surface regularity: The surface regularity (S_R) is a dimensionless ratio between the segmented tumor volume and the volume that a spherical tumor with the same surface would have.

$$S_R = 6\sqrt{\pi}\frac{Total\,Volume}{\sqrt{(Total\,surface)^3}}$$

This parameter is bounded between 0, ('complex' tumors with very irregular surfaces) and 1 (spherical tumors).

Maximum 3D tumor diameter *(d_{max} 3D)***:** It was computed as the maximal distance between two points located on the surface of the contrast enhancing tumor and provides the largest longitudinal measure of the tumor. This parameter gives a better size estimate than the frequently used maximal major axis length, based on an orthogonal set of axes.

Textural measures considered

Gradient based features: the gradient-based measures considered in this study were the spatial energies [A22], which are based on absolute gradients obtained from gray-level variations within the tumor. They are computed as the sum of all the spatial grey-level gradient variations within the segmented tumor. The spatial gray-level gradient is a vector computed on every tumor voxel by computing the differences between the grey-level values of adjacent voxels in 3D. Two different energies were computed. The spatial energy (SE) is independent of the maximum grey-level of the images, as it is normalized by the norm of all the SUV levels within the tumor. The total energy (TE) is normalized by the maximum grey-level present in the images, accounting for the spatial variations of the grey-levels within its range of values. Different energies can be computed by varying the norm with which it is computed, reflecting different physical characteristics. In this work, we used the norms 2, 3, 4, 5, 10 and 50, so obtaining 12 different energies.

Co-occurrence matrix (CM) features: the CM describe the arrangements of pairs of elements (voxels) within 2D images [A41,A42]. As it measures only relations between two voxels at a time, it is usually considered to provide information on the local texture of images. The CM was constructed by including the relationships between voxels in all of the 13 possible directions in 3D [A44,A23,28] taking only adjacent voxels. Thus, the

relations with the 26 neighbors of each voxel in 3D were considered. 5 CM-based features were considered in this work (see Table S3).

Run-length matrix (RLM) features: the RLM characterizes large areas within the tumor (groups of voxels) to provide information of regional heterogeneity [A40,A43]. Each cell in RLMs (i,j) was computed as the number of runs of length j formed by voxels of intensity in box i in all the 13 possible directions in 3D. 11 RLM-based features were considered in this work (see Table S3).

Table S3 shows the formulae of the textural features considered in this work.

Table S3 caption. Definition of the textural features considered in this study. For co-occurrence (CM) measures CM(i,j) stands for the co-occurrence matrix, N is the number of classes of grey-levels taken (16 in this study). For run-length matrix (RLM) measures RLM(i,j) is the run-length matrix, n_r is the number of runs, N is the number of classes of grey-levels and M is the size in voxels of the largest region found. Regarding the energies, u(x,y,z) denotes the gray-level of the image in the x, y and z axis.

Type of measure	Name	Formula
Co-occurrence matrix	Entropy	$-\sum_{i=1}^{N}\sum_{j=1}^{N} CM(i,j)\cdot \ln\left[CM(i,j)\right]$
Co-occurrence matrix	Homogeneity	$\sum_{i=1}^{N}\sum_{j=1}^{N} \frac{CM(i,j)}{1+(i-j)^2}$
Co-occurrence matrix	Contrast	$\sum_{i=1}^{N}\sum_{j=1}^{N} CM(i,j)\cdot (i-j)^2$
Co-occurrence matrix	Dissimilarity	$\sum_{i=1}^{N}\sum_{j=1}^{N} CM(i,j)\cdot \left\|i-j\right\|$

Co-occurrence matrix	Uniformity	$\sum_{i=1}^{N}\sum_{j=1}^{N}\left[CM(i,j)\right]^2$
Run-length matrix	Long Run Emphasis (LRE)	$\frac{1}{n_r}\sum_{i=1}^{N}\sum_{j=1}^{M}RLM(i,j)\cdot j^2$
Run-length matrix	Short Run Emphasis (SRE)	$\frac{1}{n_r}\sum_{i=1}^{N}\sum_{j=1}^{M}\frac{RLM(i,j)}{j^2}$
Run-length matrix	Low Grey-level Run Emphasis (LGRE)	$\frac{1}{n_r}\sum_{i=1}^{N}\sum_{j=1}^{M}\frac{RLM(i,j)}{i^2}$
Run-length matrix	High Grey-level Run Emphasis (HGRE)	$\frac{1}{n_r}\sum_{i=1}^{N}\sum_{j=1}^{M}RLM(i,j)\cdot i^2$
Run-length matrix	Short Run Low Grey-level Emphasis (SRLGE)	$\frac{1}{n_r}\sum_{i=1}^{N}\sum_{j=1}^{M}\frac{RLM(i,j)}{i^2\cdot j^2}$
Run-length matrix	Short Run High Grey-level Emphasis (SRHGE)	$\frac{1}{n_r}\sum_{i=1}^{N}\sum_{j=1}^{M}\frac{RLM(i,j)\cdot i^2}{j^2}$
Run-length matrix	Long Run Low Grey-level Emphasis (LRLGE)	$\frac{1}{n_r}\sum_{i=1}^{N}\sum_{j=1}^{M}\frac{RLM(i,j)\cdot j^2}{i^2}$
Run-length matrix	Long Run High Grey-level Emphasis (LRHGE)	$\frac{1}{n_r}\sum_{i=1}^{N}\sum_{j=1}^{M}RLM(i,j)\cdot i^2\cdot j^2$
Run-length matrix	Grey-Level Non-Uniformity (GLNU)	$\frac{1}{n_r}\sum_{i=1}^{N}\left(\sum_{j=1}^{M}RLM(i,j)\right)^2$
Run-length matrix	Run-Length Non-Uniformity (RLNU)	$\frac{1}{n_r}\sum_{j=1}^{M}\left(\sum_{i=1}^{N}RLM(i,j)\right)^2$

Run-length matrix	Run Percentage (RPC)	$\frac{n_r}{\sum_{i=1}^{N}\sum_{j=1}^{M} RLM(i,j)\cdot j}$
Spatial energy	Spatial energy (norm p)	$\frac{\left(\int_z \int_y \int_x \lvert\nabla u(x,y,z)\rvert^p \,\Delta x\,\Delta y\,\Delta z\,dx\,dy\,dz\right)^{\frac{1}{p}}}{\left(\int_z \int_y \int_x \lvert u(x,y,z)\rvert^p \,\Delta x\,\Delta y\,\Delta z\,dx\,dy\,dz\right)^{\frac{1}{p}}}$
Total energy	Total energy (norm p)	$\frac{\left(\int_z \int_y \int_x \lvert\nabla u(x,y,z)\rvert^p \,\Delta x\,\Delta y\,\Delta z\,dx\,dy\,dz\right)^{\frac{1}{p}}}{max_{x,y,z}\, u(x,y,z)}$

Table S4 caption. Feature importance ranking of the cross-validation-based feature selection algorithm with regression trees

PARAMETER	SCORE	IMPORTANCE RANKING
Age	1163,019194	1
Surface regularity	76,29299	2
RLM16-LGRE	72,80527	3
RLM16-HGRE	65,440498	4
RLM16-SRHGE	62,068569	5
Geometric irregularity	61,718005	6
RLM16-SRLGE	59,993552	7
CE rim width	45,871033	8
CM16-UNI	43,321344	9
CE volume	42,671811	10
RLM16-RLNU	41,345162	11
Max CE rim width	40,595562	12
Q4 CE rim width	40,082047	13
RLM16-RPC	37,486504	14
CM16-ELN	35,379336	15
Geometric heterogeneity	33,523982	16
Total Surface	33,5196	17
Max diameter	32,102192	18
Total Volume	31,105335	19
EE-2	25,759104	20
Q3 CE rim width	25,225233	21
ET-3	24,952074	22
ET-2	24,695739	23
EE-4	24,582027	24
EE-3	24,368386	25
Necrotic volume	24,13397	26
CM16-HOM	24,043498	27
ET-50	22,923418	28
RLM16-SER	22,66601	29
Q2 CE rim width	22,259601	30
CM16-COM	22,177305	31
ET-4	22,077553	32
EE-50	21,768281	33
RLM16-GLNU	21,655624	34
EE-5	21,403812	35
Mean CE rim width	21,01395	36
CM16-DIS	20,030934	37
Q1 CE rim width	19,634771	38
ET-5	19,520535	39
Mode CE rim width	18,464428	40
ET-10	18,31009	41
RLM16-LRHGE	15,34957	42
EE-10	12,848465	43
RLM16-LRE	8,191706	44
RLM16-LRLGE	5,601929	45

Table S5 caption. Feature scores per component and explained variance after Principal Component Analysis (PCA). Scores higher than 0.1 are in red and those lower than -0.1 are in blue.

	**Comp.**	**Comp.**	**Comp.**	**Comp.**	**Comp.**	**Comp.**	**Comp.**	**Comp.**
**Survival**	**-0,03**	**0,00**	**-0,04**	**0,02**	**0,00**	**0,36**	**-0,01**	**-0,40**
Age	0,01	-0,03	0,00	0,09	-0,03	-0,32	0,02	0,50
Necrotic volume	0,05	0,23	-0,10	-0,21	0,11	0,19	-0,07	0,04
CE volume	0,01	0,28	0,10	-0,08	0,10	-0,11	-0,14	0,03
CE rim width	-0,05	0,14	0,26	0,14	-0,01	-0,20	-0,06	-0,01
Max CE rim width	0,01	0,21	0,22	-0,02	0,03	0,05	-0,16	-0,12
Max diameter	0,04	0,28	0,03	-0,13	0,11	-0,15	-0,09	-0,09
Q1 CE rim width	-0,07	0,03	0,31	0,07	-0,08	0,20	0,07	0,05
Q2 CE rim width	-0,07	0,05	0,33	0,13	-0,06	0,11	0,01	0,00
Q3 CE rim width	-0,06	0,07	0,33	0,14	-0,04	0,06	-0,05	-0,04
Q4 CE rim width	0,01	0,21	0,22	-0,02	0,03	0,05	-0,16	-0,12
Mean CE rim width	-0,07	0,06	0,33	0,12	-0,05	0,10	-0,01	-0,02
Mode CE rim width	-0,06	0,05	0,30	0,11	-0,05	0,12	0,09	0,07
Geometric	0,09	0,16	-0,16	-0,18	0,09	-0,05	-0,13	-0,15
Geometric irregularity	-0.06	-0.05	0.19	0.24	-0.02	-0.15	-0.04	-0.03
Total Volume	0,04	0,29	0,00	-0,16	0,12	0,04	-0,12	0,04
Total Surface	0,04	0,29	0,02	-0,13	0,11	-0,12	-0,11	-0,01
Surface regularity	-0,03	-0,12	-0,07	-0,03	0,00	0,55	0,09	0,13
EE-2	0,25	-0,03	-0,05	0,15	0,06	0,05	-0,15	0,00
EE-3	0,25	-0,02	-0,06	0,17	0,06	0,04	-0,15	-0,02
EE-4	0,25	-0,02	-0,05	0,18	0,06	0,03	-0,15	-0,03
EE-5	0,25	-0,02	-0,05	0,20	0,06	0,02	-0,15	-0,04
EE-10	0,24	0,00	-0,04	0,23	0,06	-0,02	-0,13	-0,05
EE-50	0,23	-0,01	0,00	0,23	0,05	-0,03	-0,05	-0,02
ET-2	0,23	0,12	0,06	-0,07	-0,01	0,02	0,33	0,06
ET-3	0,25	0,09	0,06	-0,03	-0,01	0,03	0,27	0,05
ET-4	0,27	0,07	0,05	0,01	-0,01	0,04	0,23	0,03
ET-5	0,27	0,05	0,04	0,04	-0,01	0,04	0,19	0,02
ET-10	0,27	0,01	0,02	0,15	0,01	0,02	0,07	-0,01
ET-50	0,24	-0,01	0,01	0,22	0,04	-0,02	-0,03	-0,02
RLM16-SER	0,09	-0,06	0,09	-0,25	0,03	0,07	-0,14	-0,13
RLM16-LRE	-0,06	0,24	-0,10	0,08	0,00	0,17	-0,17	0,33
RLM16-LGRE	0,12	0,09	-0,05	-0,07	-0,43	0,01	-0,16	-0,09
RLM16-HGRE	-0,05	-0,11	0,06	0,02	0,48	0,12	-0,04	0,03
RLM16-SRLGE	0,12	0,08	-0,05	-0,08	-0,42	0,02	-0,20	-0,11
RLM16-SRHGE	-0,01	-0,12	0,08	-0,05	0,47	0,12	-0,09	-0,01
RLM16-LRLGE	0,00	0,15	-0,10	0,08	-0,06	0,29	-0,20	0,37
RLM16-LRHGE	-0,09	0,22	-0,04	0,07	0,11	-0,02	-0,05	0,29
RLM16-GLNU	0,16	0,18	0,07	-0,16	0,01	0,01	0,31	0,03
RLM16-RLNU	0,18	0,10	0,11	-0,22	0,07	-0,01	0,29	0,03
RLM16-RPC	0,12	-0,16	0,14	-0,20	-0,15	0,04	-0,17	0,18
CM16-ELN	0,14	-0,18	0,16	-0,21	0,07	-0,02	-0,11	0,03
CM16-HOM	-0,13	0,22	-0,16	0,17	-0,02	0,04	0,17	-0,07
CM16-COM	0,13	-0,17	0,15	-0,22	-0,09	0,08	-0,13	0,22
CM16-DIS	0,14	-0,20	0,16	-0,21	-0,05	0,03	-0,15	0,15
CM16-UNI	-0,08	0,19	-0,15	0,14	-0,11	0,24	0,08	0,07
**EXPLAINED**	**11,93**	**8,93**	**7,16**	**3,78**	**3,05**	**1,60**	**1,47**	**1,29**
